# Supplementary material for: Towards estimating the number of strains that make up a natural bacterial population
Source: Nat Commun. 2024 Jan 16;15:544. doi: 10.1038/s41467-023-44622-z (PMC10791622; doi:10.1038/s41467-023-44622-z)
Supplement: Supplementary file 1 — Supplementary Information [file 41467_2023_44622_MOESM1_ESM.pdf]

Supplemental Information for article:

Towards estimating the number of strains that make up a natural bacterial population

Tomeu Viver[1,2], Roth E. Conrad[3], Luis M. Rodriguez-R[4], Ana S. Ramírez[5], Stephanus N. Venter[6], Jairo Rocha-Cárdenas[7], Mercè Llabrés[7], Rudolf Amann[2], Konstantinos T. Konstantinidis[3], Ramon Rosselló-Móra[1]

Running Title: How many strains make up a bacterial population?

Affiliations

[1] Marine Microbiology Group, Department of Animal and Microbial Biodiversity, Mediterranean Institute for Advanced Studies (IMEDEA, CSIC-UIB), Esporles, Spain.

[2] Department of Molecular Ecology, Max Planck Institute for Marine Microbiology, Bremen, Germany.

[3] School of Civil and Environmental Engineering, and School of Biological Sciences, Georgia Institute of Technology, Atlanta, GA, USA.

[4] Department of Microbiology, and Digital Science Center (DiSC), Universität Innsbruck, Innsbruck, Tyrol, Austria.

[5] Unidad de Epidemiología y Medicina Preventiva, IUSA, Facultad de Veterinaria, Universidad de Las Palmas de Gran Canaria, C/Trasmontaña s/n, Arucas, 35413, Canary Islands, Spain.

[6] Department of Biochemistry, Genetics and Microbiology, and Forestry and Agricultural Biotechnology Institute (FABI), University of Pretoria, Pretoria, South Africa.

[7] Department of Mathematics and Computer Science, University of the Balearic Islands, Palma 07122, Spain.

Supplementary Note 1: Genomic differences between isolates of distinct CVs.

All members of the same CV consistently exhibited ANI >99.99%, but we also observed a few cases of genomes belonging to different RAPD categories that showed high ANI values. For example, CV3 isolates (DW07 and DW11), despite being distinct based on RAPD4, showed highly similar RAPD6 profiles with isolates DW08 and DW10 obtained from the same sample. Moreover, RAPD5 discriminated between CV3 (DW07 and DW11) and both DW08 and DW10, which shared identical pattern (Figure S9). Consistently, genomes from CV3 displayed ANI values >99.96% with genomes DW08 and DW10, and an average shared genome fraction of 91% with DW08 and 93% with DW10. Genomes DW08 and DW10 displayed an ANI value of 99.94% and shared genome fraction of 95.4% between them (Sup. Data S7). The pangenome of the four isolates indicated that the genomes from CV3 encoded for an identical set of 3,208 genes of which 258 were specific to CV3. 131 genes were specific to DW08 and 64 genes to DW10. Most strain-specific genes were encoded in a single contig in each respective assembly, indicating that they represent a genome island (e.g., a plasmid or a prophage). The functional annotation of CV3 genomes showed that among the 258 CV-specific genes there were a phage integrase, an intron reverse transcriptase, a DNA helicase, two different CRISPR Cas systems (one of them associated to CRISPR type I-E), a plasmid partition protein (ParA) and an environmental halophage (Sup. Data S8). In genome DW10, the genome-specific genes encoded for a ParA, 5 transposases, 1 integrase, 1 helicase, and a pilin glycosylation enzyme, in addition to hypothetical and other poorly characterized proteins. In genome DW08, the genome-specific genes encoded for viral genes (as VirE and nucleases), phage genes Resolvase/invertase-type recombinase genes and three different contigs encoded for ParA genes.

Similarly, CV5 and CV6 isolates showed nearly identical RAPD profiles using the three set of primers. However, isolates from CV6 presented a genome length ~80,000 bps larger than those of CV5. Pan-genome analysis comparing these two CVs revealed that over 99.5% of the CV5 genes were shared with CV6 isolates. Specifically, CV6 carried 56 additional genes not carried by CV5. Analyzing the metabolic functions in the FV16 genome (CV6), we detected that these genes were carried by two contigs (contig 22 and 28; Sup. Data S9); whereas the corresponding genes were in the same contig in genome FV68. In the two contigs, we detected the presence of CRISPR spacers. Contig 22 encoded for 2 nucleases, a DNA polymerase, 2

transposases, a DNA invertase, a recombinase and an endonuclease. Contig 28 encoded one ParA family protein, a RepB plasmid replication protein, and one CRISPR Cas system (Cas2, Cas1, Cas4, Cas7, Cas3, Cas6 proteins). The gene annotation indicated that the contigs belong to a plasmid and the coverage was two times higher than the chromosome indicating a double-copy plasmid. Altogether, these results showed that the most closely related CVs had substantial gene content differences (in addition to their ANI dissimilarities), albeit typically smaller than those observed between most CVs sharing ~98% ANI, that could underlie ecological differences and/or adaptations such as phage predation.

Supplementary Figure S1: Identification of *Sal. ruber* clonal varieties (CVs) using RAPD signatures. Identical RAPD profiles were interpreted to belong to the same CV. RAPD fingerprints were obtained using RAPD4, RAPD5 and RAPD6 amplification primers[1]. Each column in the gels shows a RAPD profile for a different isolate. Isolates of CV1 to CV4 were retrieved from a mesocosm experiment from the Mallorca solar salterns (CZ: Control pond time zero; DW: Dilution pond time 1 week; UZ: Unshaded pond time zero). The mesocosm experiments were described previously[2,3,4]. Isolates of CV5 and CV6 were retrieved from Fuerteventura solar salterns.

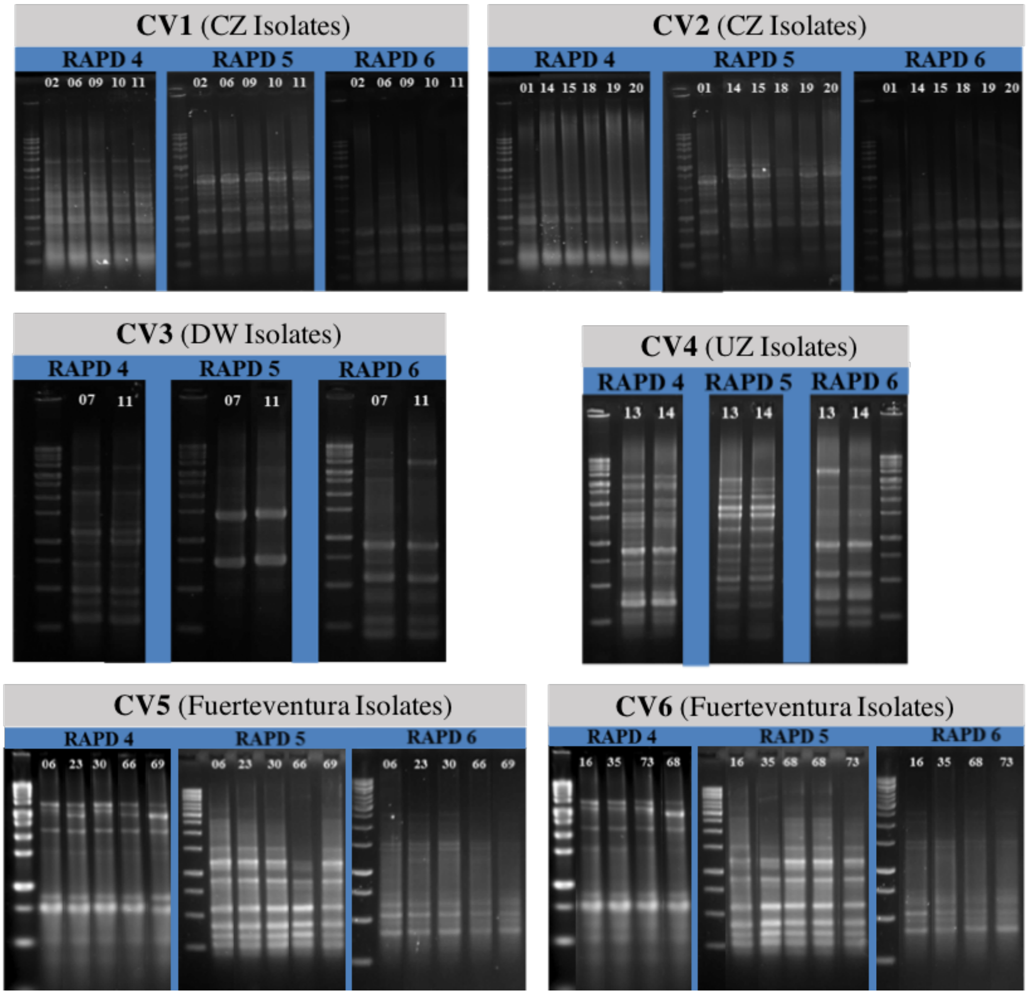

Supplementary Figure S2: Genomic diversity among *Sal. ruber* genomes recovered from the individual mesocosm experiments in the Mallorca solar salterns. The ANI value between pairs of genomes (x-axis) from the same mesocosm (graph title) is plotted against their shared genome fraction (y-axis). The graph to the top of each panel shows the number of ANI comparisons in each range of ANI value (0.1%). The mesocosm experiments were described previously[2,3,4].

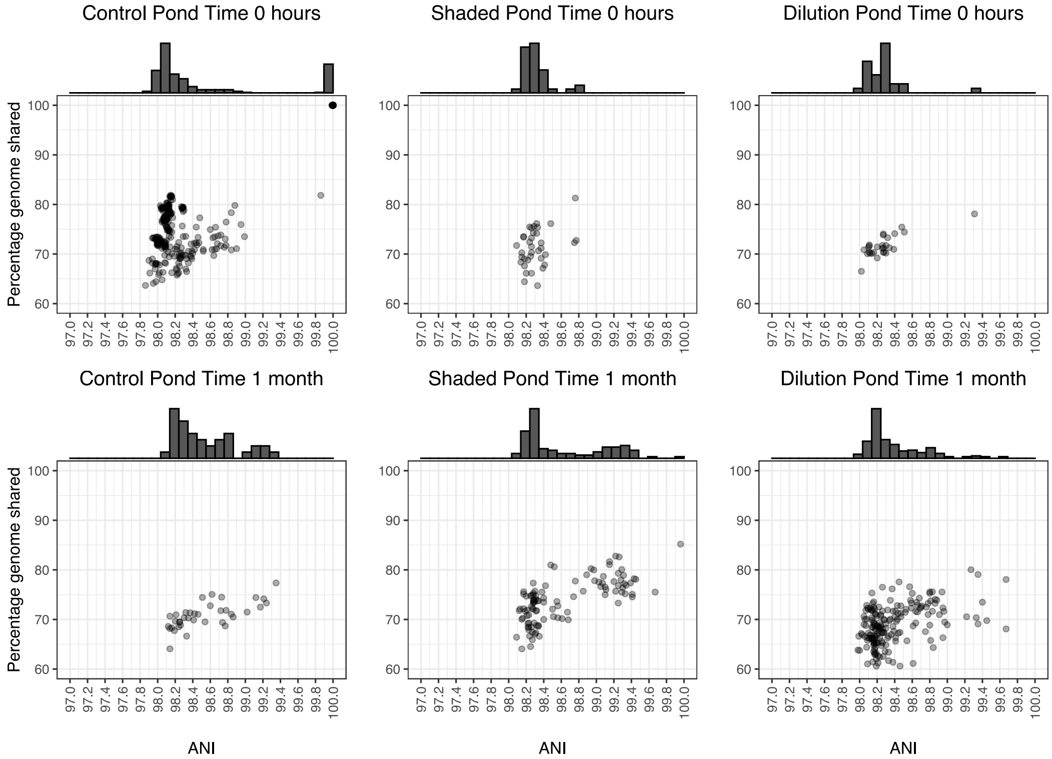

Supplementary Figure S3: Bootstrap resampling analysis on the kernel density estimates to identify variation in local minimum and maximum regions of the ANI distribution. We performed a bootstrap resampling analysis to produce estimates and confidence intervals for local minimum and maximum ranges in the ANI distribution as described in detail in the Methods section. Briefly, we performed 10,000 bootstrap iterations, and for each iteration, we randomly sampled with replacement the entire dataset of ANI values, computed the kernel density estimate across the new distribution (scipy.stats.gaussian_kde, bw_method=0.15), and identified local minimums and maximums (scipy.signal.find_peaks, default settings). The top panel shows the empirical distribution for all data (all iterations combined) with the local minimum identified in the range within 99.6% to 99.8% ANI. The second panel shows the results of a single bootstrap iteration and the third panel shows the results from all 10,000 bootstrap iterations with the 95% confidence interval surrounding the mean kernel density estimate in blue. The density and spread of local minimum and maximum values are marked with dark gray or light gray vertical lines in the third panel as well. The bottom panel shows minimum and maximum results from the third panel as a histogram. Note that the empirical distribution in the top panel is far from a Gaussian or Uniform distribution as there is clear deviation from the mean bin count across the ANI distribution. Note also that the clearly observed minimum around 99.6% to 99.8% ANI is highly stable and consistent with what is reported in the main text.

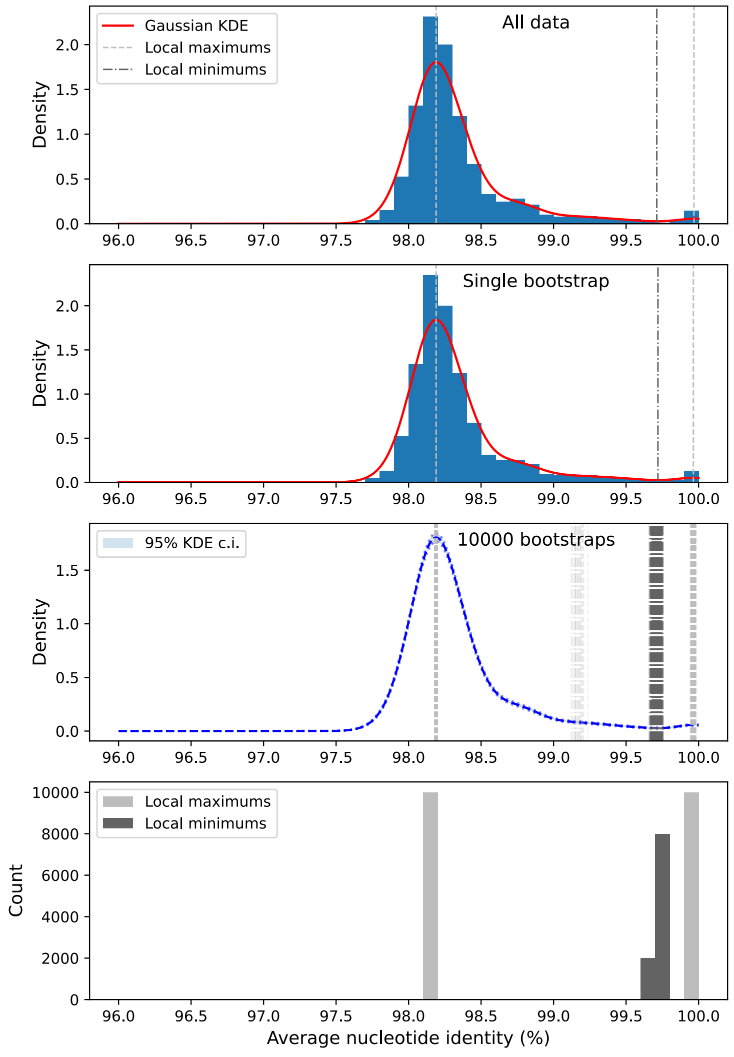

Supplementary Figure S4: ANI value distribution of *Sal. ruber* genomes based on 793 core ortholog genes. The histogram shows the number of datapoints for x-axis (in 0.1% windows or bins). Contrasting this graph to the main graph shown in Figure 1 shows that ANI values calculated based on the whole genome is similar to that based on core genes only (this Figure). Source data are provided as a Source Data 4.

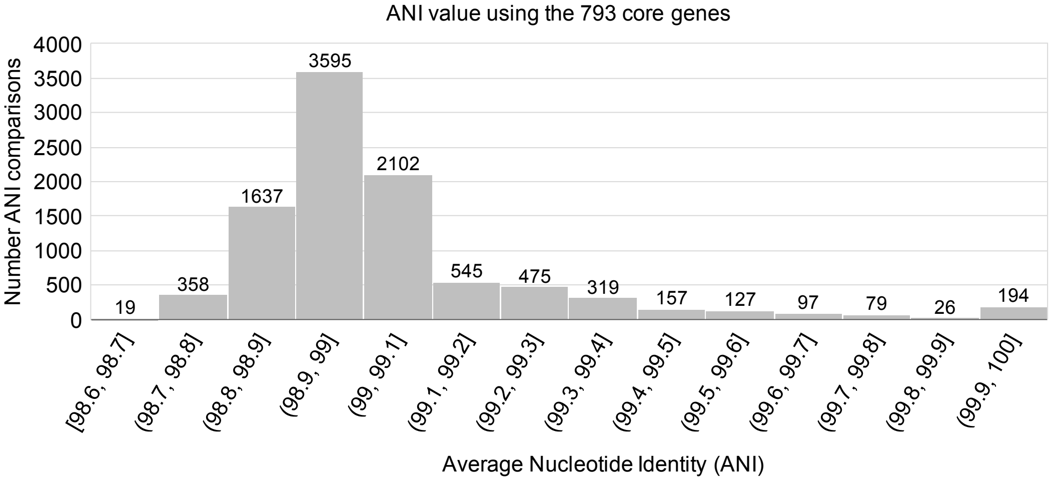

Supplementary Figure S5: Bootstrap resampling analysis to identify variation in local minimum and maximum regions of the ANI distribution using all *Sal. ruber* genomes from this study and from public databases (211 genomes). We performed a bootstrap resampling analysis to produce estimates and confidence intervals for local minimum and maximum ranges in the ANI distribution as described in detail in the Methods section. Briefly, we performed 10,000 bootstrap iterations, and for each iteration, we randomly sampled with replacement the entire dataset of ANI values, computed the kernel density estimate across the new distribution (scipy.stats.gaussian_kde, bw_method=0.15), and identified local minimums and maximums (scipy.signal.find_peaks, default settings). The top panel shows the empirical distribution for all data (all iterations combined) with the local minimum identified in the range within 99.6% to 99.8% ANI. The second panel shows the results of a single bootstrap iteration and the third panel shows the results from all 10,000 bootstrap iterations with the 95% confidence interval surrounding the mean kernel density estimate in blue. The density and spread of local minimum and maximum values are marked with dark gray or light gray vertical lines in the third panel as well. The bottom panel shows minimum and maximum results from the third panel as a histogram. Note that the empirical distribution in the top panel is far from a Gaussian or Uniform distribution as there is clear deviation from the mean bin count across the ANI distribution. Note also that the clearly observed minimum around 99.6% to 99.8% ANI is highly stable and consistent with what is reported in the main text and in Figure 1 based only on the genomes reported by our study. Source data are provided as a Source Data 5.

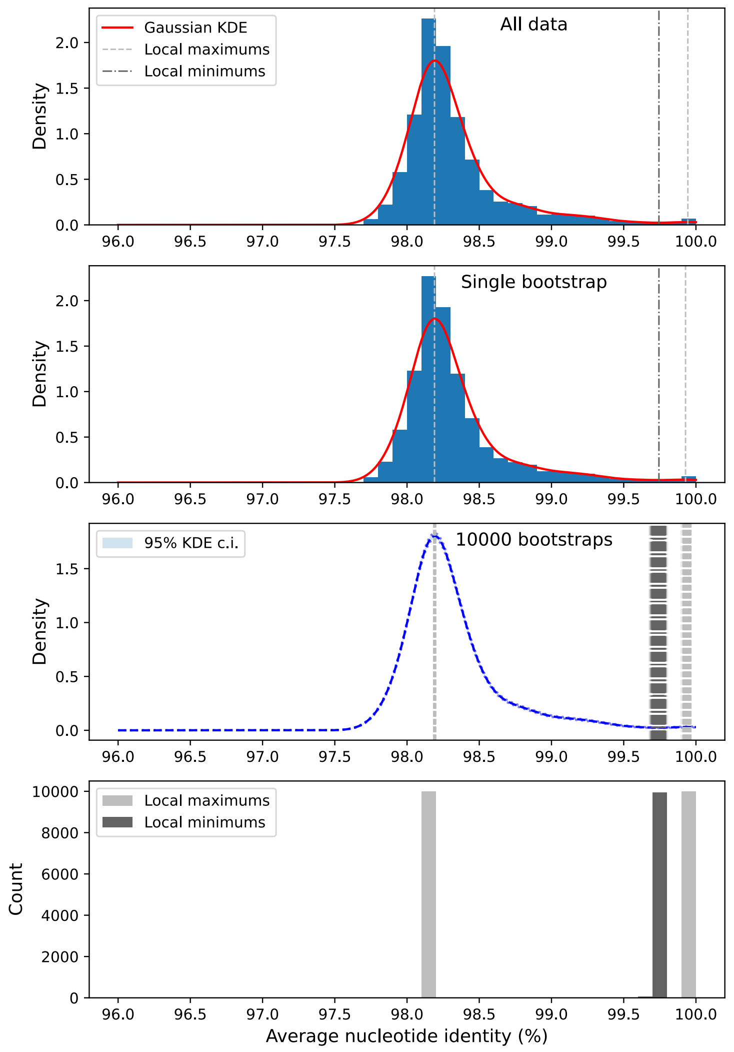

Supplementary Figure S6: Genomic diversity assessed by the number of different alleles within each core-orthology group (OG) identified. 793 OGs were assessed.

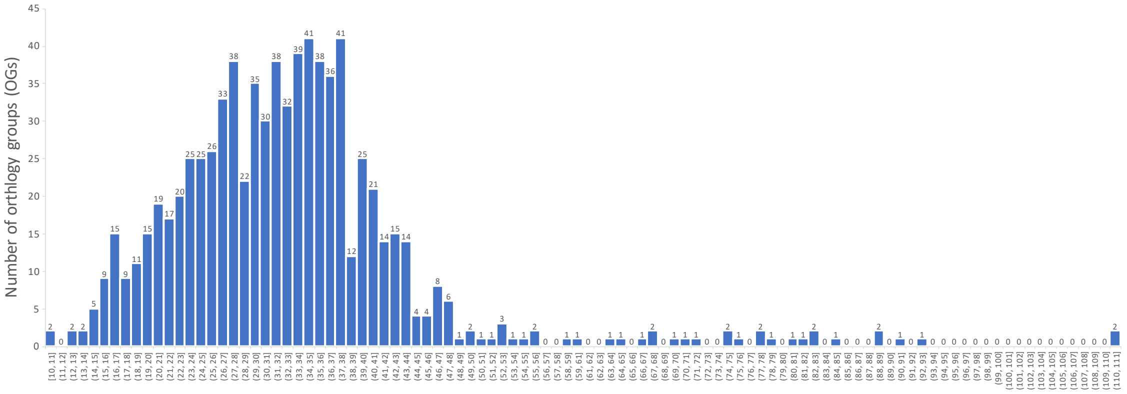

Supplementary Figure S7: Genomic clustering of isolate genomes based on the percentage of alleles shared. In the analysis, 793 core-OGs without paralogs (i.e., single-copy) were included. Alleles were defined as genes sharing at least 99.8% nucleotide identity. The percentage of alleles shared between genomes at this identity level was used as a distance metric to cluster the genomes, and the resulting overall similarity in gene-content was represented in a heatmap using the ggplot2 package v3.3.6 in Rstudio v1.1.4. Source data provided in Supplementary data S2.

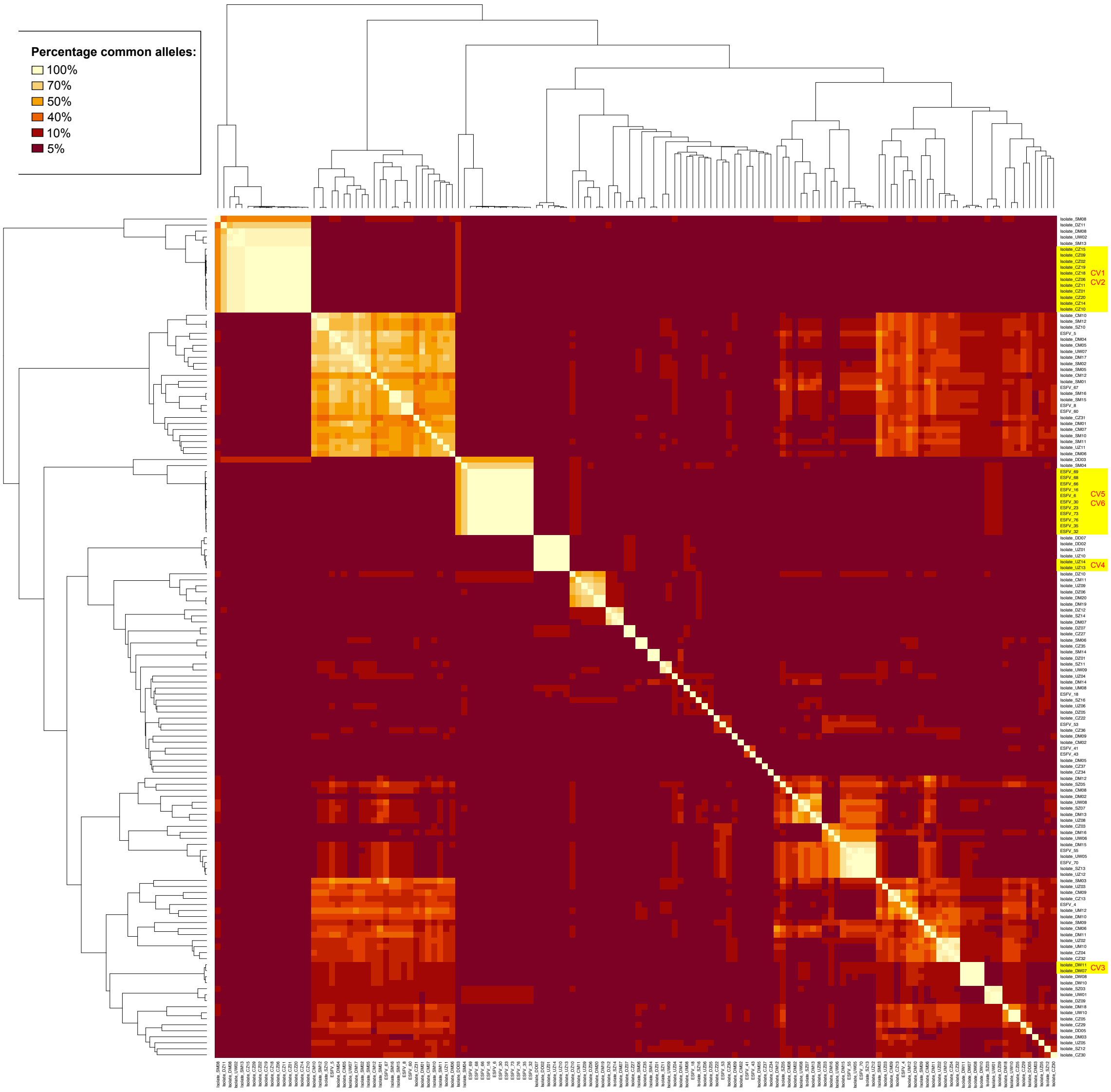

Supplementary Figure S8: Comparison between genomic clustering based on the percentage of alleles shared (left) and phylogenetic analysis based on 793 core ortholog genes (right). To identify allelic variation in core genes among the genomes, the sequences assigned to a single OG were grouped using a 99.8% nucleotide sequence identity cut-off (see also Results section for further details). The percentage of alleles shared between genomes at this identity level was used as a distance metric to cluster the genomes, and the resulting dendrogram (on the left) is compared to a phylogenetic tree based on the 793 core genes using Neighbor Joining algorithm (on the right). In both the dendrogram and phylogenetic tree, genomes belonging to the same genomovar (GV) were highlighted in grey boxes and a vertical black line. In the phylogenetic tree, those genomes showing a higher abundance under high-salt conditions were marked with a red circle, while those genomes with higher abundance under low-salt conditions were marked with a blue circle.

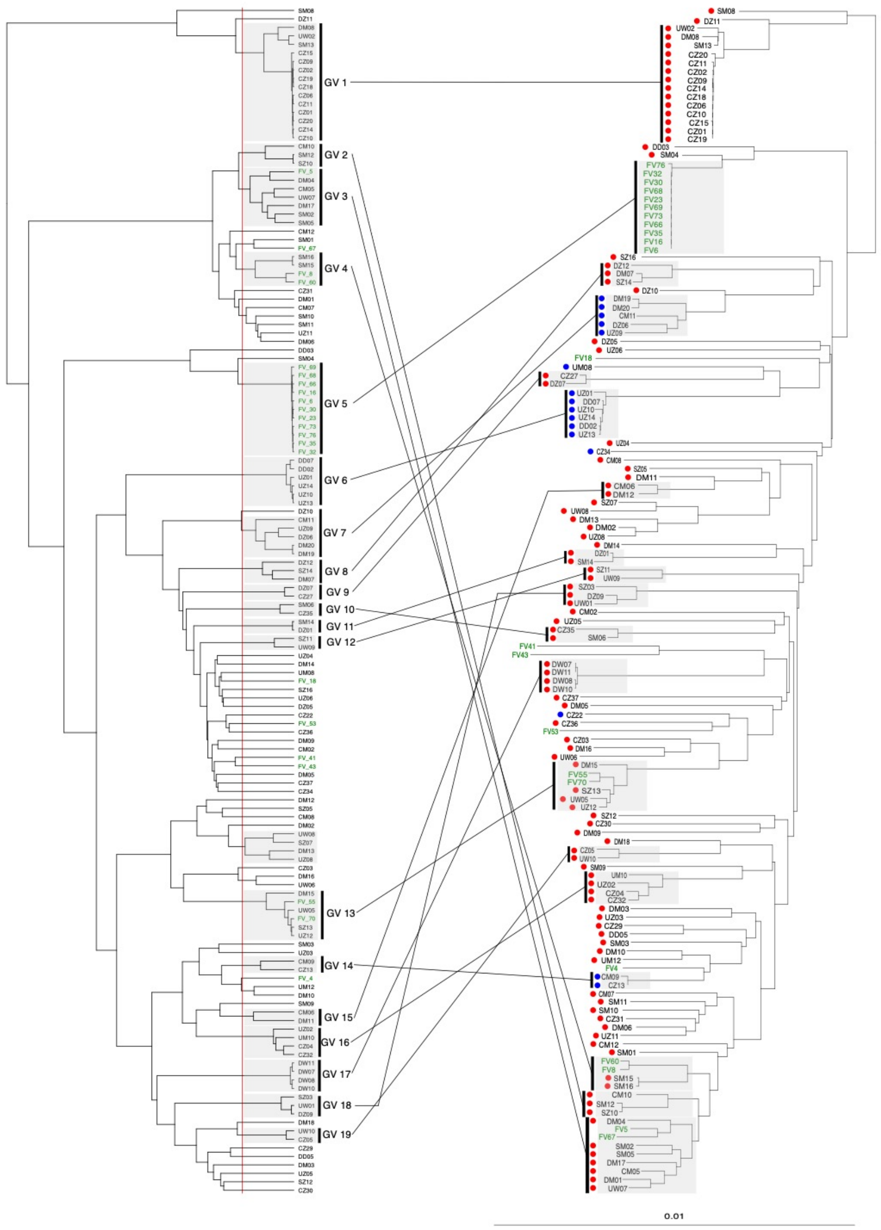

Supplementary Figure S9: RAPD signatures of CV3 and comparison with their close relative isolates. RAPD fingerprints were obtained using the RAPD4, RAPD5 and RAPD6 amplification primers[1]. Each column in the gels shows a RAPD profile for a different isolate. Isolates DW07 and DW11 belong to the same CV, CV3, and are designated by blue fonts; closely related isolates are designated by other colors.

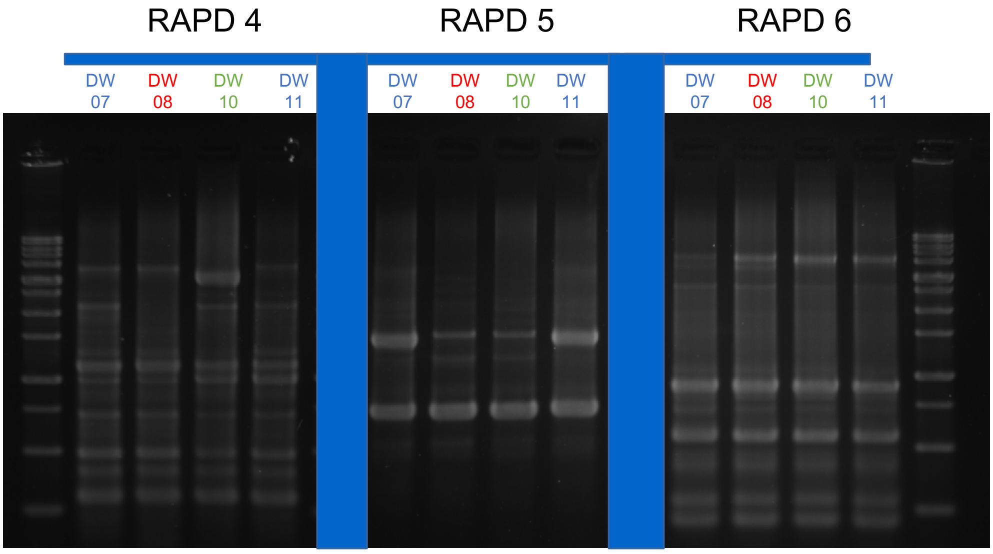

Supplementary Table S1: Nonpareil analysis of the metagenomic reads representing the total *Sal. ruber* population in the control pond over a sampling period of one month.

Nonpareil Statistics	Time zero	Time 1 week	Time 1 month	Concatenation of the three samples
Redundancy	0.927	0.953	0.941	0.975
Average coverage	0.933	0.957	0.946	0.977
Actual sequencing effort	47,651,918	74,515,635	50,224,608	172,392,831
Pearson's coefficient	0.999	0.999	0.999	0.999
Estimated sequencing effort	45,957,814	40,539,528	39,110,605	39,934,906
Nonpareil sequence-diversity index	14.58	14.50	14.47	14.49

Supplementary Table S2: Estimations of the number of genomovars making up the total *Sal. ruber* population and 99% prediction interval (lower and upper boundaries).

Sample (Control Pond)	Strains fit	Strains lower	Strains upper
Time zero (CZT0h)	11,113	9,205	13,416
Time 1 week (CZT1W)	6,181	5,060	7,549
Time 1 month (CZT1M)	5,496	4,501	6,711
Concatenation of the three samples	6,775	5,605	8,190

SUPPLEMENTARY REFERENCES

1. Sikorski, J., Rosselló-Mora, R. & Lorenz, M. G. Analysis of genotypic diversity and relationships among *Pseudomonas stutzeri* strains by PCR-based genomic fingerprinting and multilocus enzyme electrophoresis. *Syst. Appl. Microbiol*. **22**, 393-402 (1999).
2. Viver, T. *et al*. Distinct ecotypes within a natural haloarchaeal population enable adaptation to changing environmental conditions without causing population sweeps. *ISME J*. **15**, 1178-1191 (2021).
3. Viver, T. *et al*. Predominance of deterministic microbial community dynamics in salterns exposed to different light intensities. *Environ. Microbiol*. **21**, 4300-4315 (2019).
4. Conrad, R. E. *et al*. Toward quantifying the adaptive role of bacterial pangenomes during environmental perturbations. *ISME J*. **16**, 1222-1234 (2022).
