# Supplementary material for: Towards estimating the number of strains that make up a natural bacterial population
Source: Nat Commun. 2024 Jan 16;15:544. doi: 10.1038/s41467-023-44622-z (PMC10791622; doi:10.1038/s41467-023-44622-z)
Supplement: Supplementary file 3 — Description of Additional Supplementary Files [file 41467_2023_44622_MOESM3_ESM.pdf]

Description of Additional Supplementary Files:

Supplementary Data 1: Genomic characteristics of the subsampled genomes.

Supplementary Data 2: Percentage of alleles shared between pair of genomes

Supplementary Data 3: Metagenomic relative abundance of the isolates representing different genomovars. The table shows the relative abundance of the isolates based on metagenomic reads from the sample where the isolates were recovered. Relative abundance was estimated based on a competitive best-match approach to map reads to core genes as described in the main text. (A) Mallorca isolates (rows) and metagenomes (columns); (B) Fuerteventura isolates (rows) and metagenomes (columns).

Supplementary Data 4: Genomic characteristics of the genomes associated to CVs.

Supplementary Data 5: Genomic similarities between genomes from CV1 and CV2

Supplementary Data 6: Genomic characteristics of the genomes from isolates

Supplementary Data 7: ANI values and percentage of shared genomic fragments between genomes belonging to the same sample but different CV. ANI values between genomes are given in the lower triangle. The percentage of shared genomic fragments are given in the upper triangle.

Supplementary Data 8: Strain specific gene annotation of DW07 (CV2), DW08 and DW10 and indemnification of the genes in other genomes from the Salinibacter ruber genome collection

Supplementary Data 9: Functional prediction of genes carried by the plasmid detected in CV6
