# Supplementary material for: Towards estimating the number of strains that make up a natural bacterial population
Source: Nat Commun. 2024 Jan 16;15:544. doi: 10.1038/s41467-023-44622-z (PMC10791622; doi:10.1038/s41467-023-44622-z)
Supplement: Supplementary file 5 — Reporting Summary [file 41467_2023_44622_MOESM5_ESM.pdf]

nature portfolio

Corresponding author(s): Konstantinos T. Konstantinidis
Ramon Rosselló-Móra

Last updated by author(s): Dec 5, 2023

Reporting Summary

Nature Portfolio wishes to improve the reproducibility of the work that we publish. This form provides structure for consistency and transparency in reporting. For further information on Nature Portfolio policies, see our Editorial Policies and the Editorial Policy Checklist.

Statistics

For all statistical analyses, confirm that the following items are present in the figure legend, table legend, main text, or Methods section.

n/a	Confirmed	
☐	☒	The exact sample size (*n*) for each experimental group/condition, given as a discrete number and unit of measurement
☒	☐	A statement on whether measurements were taken from distinct samples or whether the same sample was measured repeatedly
☐	☒	The statistical test(s) used AND whether they are one- or two-sided<br>*Only common tests should be described solely by name; describe more complex techniques in the Methods section.*
☒	☐	A description of all covariates tested
☐	☒	A description of any assumptions or corrections, such as tests of normality and adjustment for multiple comparisons
☐	☒	A full description of the statistical parameters including central tendency (e.g. means) or other basic estimates (e.g. regression coefficient) AND variation (e.g. standard deviation) or associated estimates of uncertainty (e.g. confidence intervals)
☐	☒	For null hypothesis testing, the test statistic (e.g. *F*, *t*, *r*) with confidence intervals, effect sizes, degrees of freedom and *P* value noted<br>*Give P values as exact values whenever suitable.*
☒	☐	For Bayesian analysis, information on the choice of priors and Markov chain Monte Carlo settings
☒	☐	For hierarchical and complex designs, identification of the appropriate level for tests and full reporting of outcomes
☐	☒	Estimates of effect sizes (e.g. Cohen's *d*, Pearson's *r*), indicating how they were calculated

Our web collection on statistics for biologists contains articles on many of the points above.

Software and code

Policy information about availability of computer code

Data collection	No softwares were used for data collection
Data analysis	BBDuk v38.82, SPADES v3.14, Prodigal v2.6.3, SwissProt an TrEMBL databases, Diamond v0.9.31, Mauve v2.4.0, BLASTn v2.2.28, ARB v6.0.6, Nonpareil v3.03, R package stats v4.0.3. ani.rb, ogs.mcl.rb, Aln.cat.rb, HMM.essential.rb BlastTab.best_hit_sorted.pl scripts from the Enveomics script collection (available at http://enve-omics.gatech.edu).<br>The code for ANI peak finding and the kernel density estimate can be found at https://github.com/rotheconrad/bacterial_strain_definition.<br>The code for estimate the number of genomovars is availale at https://github.com/TomeuViver/Estimations-genomovars.

For manuscripts utilizing custom algorithms or software that are central to the research but not yet described in published literature, software must be made available to editors and reviewers. We strongly encourage code deposition in a community repository (e.g. GitHub). See the Nature Portfolio guidelines for submitting code & software for further information.

Data

Policy information about availability of data

All manuscripts must include a data availability statement. This statement should provide the following information, where applicable:

- Accession codes, unique identifiers, or web links for publicly available datasets
- A description of any restrictions on data availability
- For clinical datasets or third party data, please ensure that the statement adheres to our policy

The metagenomes from Mallorca and Fuerteventura solar salterns were deposited in the European Nucleotide Archive (ENA) under BioProject accession numbers PRJEB27680 and PRJEB45291, respectively. All genomes included in the study were also deposited in ENA and listed in Supplementary Spreadsheet S5.

Research involving human participants, their data, or biological material

Policy information about studies with human participants or human data. See also policy information about sex, gender (identity/presentation), and sexual orientation and race, ethnicity and racism.

Reporting on sex and gender	Not applicable
Reporting on race, ethnicity, or other socially relevant groupings	Not applicable
Population characteristics	Not applicable
Recruitment	Not applicable
Ethics oversight	Not applicable

Note that full information on the approval of the study protocol must also be provided in the manuscript.

Field-specific reporting

Please select the one below that is the best fit for your research. If you are not sure, read the appropriate sections before making your selection.

☐ Life sciences ☐ Behavioural & social sciences ☒ Ecological, evolutionary & environmental sciences

For a reference copy of the document with all sections, see nature.com/documents/nr-reporting-summary-flat.pdf

Ecological, evolutionary & environmental sciences study design

All studies must disclose on these points even when the disclosure is negative.

Study description	In this study we have developed a genomic comparison of 138 Salinibacter ruber genomes recovered from Mallorca and Fuerteventura solar salterns, also including their comparison with short-reads metagenomes from the same samples. The genomic comparison using the Average Nucleotide Identity (ANI) values revealed a natural "gap" in space, with lower ocurrence of ANI values between 99.8% and 99.6% identity. We used this ANI gap to define genomovars and a higher ANI value of >99.99% and shared gene-content >99.0% to define strains. Moreover, using the metagenomic dataset, we extrapolated that within one natural sample, the total population is composed of 5,500 to 11,000 genomovars.
Research sample	The research samples included in this study were from (i) Mallorca solar salterns, in which the genomes and metagenomes were published in Conrad et al., 2022, and (ii) Fuerteventura solar salterns, located in the Canary Islands, from where we included the genomes and metagenome from a single sample collected in year 2019. Moreover, we included all Salinibacter ruber genomes available from public databases (i.e., NCBI and JGI). The metagenomes represent the complete microbial populations inhabiting in the analyzed ponds. The genomes represent the Salinibater ruber population.
Sampling strategy	We did not applied statistical methods to predetermine the sampling size. The metagenomes used for this study were collected in Conrad et al., 2022, in where we developed a mesocosmos study in Es Trenc solar saltern (Mallorca) in three continuous ponds. We collected samples at the begining of the experiment and then, at 1 week and after 1 month. In the Fuerteventura solar saltern we just collected one sample from a single crystallyzer pond. Samples were manually collected using 1 L jars. The samples were collected in a temporal series of one month to enhance the statistics analysis.
Data collection	The samples were collected by the members of the Marine Microbiology Group of the IMEDEA. Samples were collected in sterile bottles and transported to the IMEDEA facilities for further manipulation.
Timing and spatial scale	The mesocosms experiments in Es Trenc solar saltern were developed in year 2012, as reported in Viver et al., 2019, Viver et al., 2022 and Conrad et al., 2022. The samples in Es Trenc solar salterns were collected from 1st to 31th August 2012, collecting samples in time zero, one week and one month. The sample from Fuerteventura was collected in July 2019.
Data exclusions	No data was excluded from the analysis

Reproducibility	For the field sample replicates were not considered due to elevated costs in sampling, metagenome and genome sequencing, and no big need due to time-series scheme. For the bioinformatic reproducibility, our code is freely available and the Methods section include all details to reproduce the bioinformatics results.
Randomization	In this study, the "culture" process is based on random selection, with the exception of the final selection of isolates for genome sequencing where we intentionally opted to not sequence many representatives of the same Clonal Variety (CVs) based on RAPD profiles to avoid sequencing many very similar genomes. The RAPD analysis identified a total of 9 CVs with more than one representative genome and we sequence more than 1 representative from only four of them and not all representatives available but a randomly chosen subset.
Blinding	Blinding is not applicable to our study because we do not compare between groups of samples or human cohorts.

Did the study involve field work? [x] Yes [ ] No

Field work, collection and transport

Field conditions	The samples were collected from 'Es Trenc' solar salterns with permision from Gusto Mundia, S.L. (flor de sal d'Es Trenc).
Location	Solar salterns of 'Es Trenc' are located in Mallorca Island (39°22'31"N 2°59'14"E2). Solar salterns of Fuerteventura are located in Fuerteventura Island (Canary Islands - 28°21'58''N 13°52'12''W).
Access & import/export	The enterprises allowed us collect samples from the ponds. The experiment in 'Es Trenc' was conducted in year 2012 and the Nagoya Protocol permits were required. The sample from Fuerteventura was collected in July 2019 and sample was collected in accordance with the permit ESNC27, with the unique identifier ABSCH-IRCC-ES-241224-1 that has been provided by the Dirección General de Biodiversidad y Calidad Ambiental del Ministerio para la Transición Ecológica of the Spanish Government.
Disturbance	During the experiments no disturbances were caused/detected

Reporting for specific materials, systems and methods

We require information from authors about some types of materials, experimental systems and methods used in many studies. Here, indicate whether each material, system or method listed is relevant to your study. If you are not sure if a list item applies to your research, read the appropriate section before selecting a response.

Materials & experimental systems

n/a	Involved in the study
[x]	[ ] Antibodies
[x]	[ ] Eukaryotic cell lines
[x]	[ ] Palaeontology and archaeology
[x]	[ ] Animals and other organisms
[x]	[ ] Clinical data
[x]	[ ] Dual use research of concern
[x]	[ ] Plants

Methods

n/a	Involved in the study
[x]	[ ] ChIP-seq
[x]	[ ] Flow cytometry
[x]	[ ] MRI-based neuroimaging

Plants

Seed stocks	Not applicable
Novel plant genotypes	Not applicable
Authentication	Not applicable
